# Pharmacovigilance in the Era of Artificial Intelligence: Advancements, Challenges, and Considerations

**DOI:** 10.7759/cureus.86972

**Published:** 2025-06-29

**Authors:** Eli Rudnisky, Keshab Paudel

**Affiliations:** 1 Biomedical Sciences, Burrell College of Osteopathic Medicine, Melbourne, USA

**Keywords:** adverse drug reactions (adr), ai and machine learning, artificial intelligence in medicine, natural language processing (nlp), pharmacovigilance

## Abstract

Pharmacovigilance (PV) is a science that plays a crucial role in protecting patients by detecting adverse drug reactions (ADRs). PV can do this by collecting and analyzing data from a wide variety of healthcare sources. However, traditional PV methods face limitations, particularly in accurately and efficiently analyzing large datasets. This limitation leads to underreported ADRs, which negatively impact many patients. However, with the recent rise in artificial intelligence, PV as a science has the potential to improve. This can be done by incorporating different subsets of AI, such as machine learning (ML) and natural language processing (NLP), into PV. The aim of this study is to describe how integrating AI, specifically ML and NLP, into PV systems can improve data collection, data processing, and the detection of ADRs. A comprehensive literature search was conducted using PubMed and Google Scholar to examine studies that were conducted within the last 30 years. Twenty-eight studies were included in this paper. Inclusion criteria included articles that were written in English, articles focusing on PV as a science, ADRs, AI’s current role in PV, and AI’s potential role in PV. Exclusion criteria included studies that were not published in English and studies that were published more than 30 years ago. The findings from several systematic reviews that explore the implementation of AI into PV indicate that AI can improve PV by enhancing the efficiency and accuracy of detecting ADRs. Through ML algorithms, ADRs can be identified more quickly and accurately compared to traditional PV methods; while using the NLP model, AI is able to extract relevant patient data from unstructured data sources such as electronic health records (EHRs) and report certain drug interactions more accurately and efficiently. However, there are limitations to incorporating AI into PV. These include ethical, legal, and privacy concerns; interpretative limitations if certain datasets are incomplete and are missing information; the lack of current research; and the need to conduct more research on this topic to definitively determine whether AI should be incorporated into PV. With the exponential development of technology such as AI, there is a lot of promise in strengthening PV into a more accurate and efficient ADR detection system. While there is some research highlighting AI’s potential to enhance PV, much more research needs to be conducted to fully substantiate this claim. Incorporating AI into PV does, however, have the potential to change ADR detection methods for the better.

## Introduction and background

With the historic rise of artificial intelligence (AI), many traditional technological methods of collecting and analyzing data are becoming less prevalent. Using traditional statistical methods of data collection and analysis might struggle to identify patterns and associations due to the sheer amount and complexity of the data [1]. Incorporating newly developed and more sophisticated technology can potentially be used to address this issue. AI is one example of sophisticated technology that can impact many different sectors of the health field, particularly pharmacovigilance (PV). PV is the science of detecting, analyzing, interpreting, and preventing adverse effects or any other medicine-/vaccine-related problems [1]. PV is a crucial science because its main purpose is to identify potential adverse drug reactions. Identifying these adverse drug reactions (ADRs) in a timely manner ensures that patients are protected [1,2]. PV requires that large amounts of data from a wide variety of sources, such as electronic health records (EHRs), FDA adverse event reporting systems (FAERS), published literature, patient registries, patient support programs, spontaneous reporting systems, and social media are collected and analyzed in a timely manner [3,4]. Analyzing large amounts of data from diverse sources and databases manually is inefficient and leads to underreported ADRs, duplication of reports in PV databases, and undetected and under-recognized drug-drug interactions, all of which potentially threaten patient safety [5-7]. The incorporation of AI systems, such as natural language processing (NLP) and machine learning (ML), has been shown to reduce time and effort in the collection and analysis of large amounts of data, can improve data quality, and can possibly increase the effectiveness of detecting ADRs [8,9].

Objective

The aim of this systematic review is to describe how integrating AI into PV can optimize ADR monitoring and improve the efficacy of gathering and interpreting large amounts of data.

Methods

**Figure 1:**
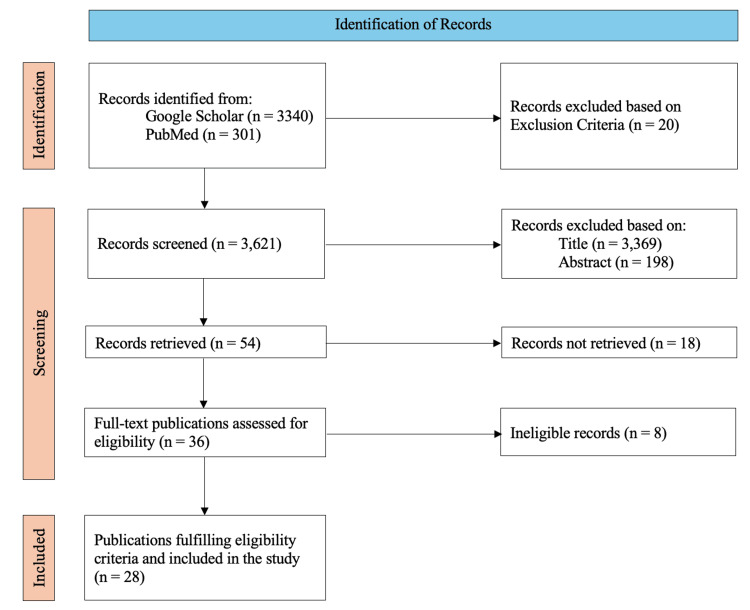
Flow chart of publications screened and identified for the systematic review PRISMA = Preferred Reporting Items for Systematic Reviews and Meta-Analyses

A comprehensive literature search was conducted using PubMed and Google Scholar to identify relevant research studies on the potential role of AI in improving PV. The purpose of the search was to find articles pertaining to the current role of AI, the impact AI has made in PV, PV as a science, the science of ADRs, and the potential risks, benefits, and limitations of the role of AI in PV. The primary search included using keywords such as “Pharmacovigilance”, “Artificial Intelligence”, “Drug Safety”, Adverse Drug Reactions”, and “Machine Learning”. These keywords were searched independently, and Boolean operators (AND, OR) were used in PubMed and Google Scholar to enhance the search. The inclusion criteria for this search were studies focusing on the current role of AI in PV, the potential role of AI in PV, PV as a science, defining what an ADR is, publications written in the English language, and papers that were published within the last 30 years. Specifically, 30 years was picked because it demonstrates how PV was utilized and viewed in the past. It also demonstrates how PV was able to mature as a science over time, especially through the incorporation of AI into PV systems. Exclusion criteria were studies that were non-English, studies that did not focus on any of the aforementioned areas of interest, and studies that were published more than 30 years ago. The Preferred Reporting Items for Systematic Reviews and Meta-Analyses (PRISMA) flow chart (Figure 1) demonstrates how relevant studies were retrieved and used for this systematic review.

## Review

Adverse drug reactions (ADRs)

ADRs are unintended and harmful responses to medications, ranging from mild to life-threatening [10]. ADRs can further be categorized into two subtypes, type A and type B reactions. Type A reactions occur as the result of known pharmacological properties, while type B reactions occur when the pharmacological properties of the drug do not predict the reaction [10]. ADRs in some countries even rank among the top 10 leading causes of mortality [11]. It is crucial that ADRs are reported in a timely and accurate manner to help prevent some of these mentioned issues.

There are multiple ways in which ADRs are reported. These methods include spontaneous reporting systems, formal drug safety studies, published data, and pharmaceutical company data [12]. PV collects and analyzes these reporting methods to help identify and report ADRs in a timely and efficient manner. For example, in a study conducted by Barry et al. [13], they stated, “Health Canada continues to release advisories of suspected adverse drug reactions that have been discovered through pharmacovigilance. These include the risk of bladder cancer with pioglitazone... and bone fractures with proton pump inhibitors, etc.” [13]. While ADRs are an inherent risk of pharmaceutical use, PV enables their timely and efficient detection and reporting.

Pharmacovigilance (PV)

PV plays a vital role in detecting ADRs and informing various sectors of the healthcare system about medication safety. It was initially developed by regulatory agencies such as the FDA to ensure safe pharmaceutical drugs for the public [14]. It consists of finding and assessing safety signals of a pharmaceutical product and comprises data accumulation related to assessing and detecting ADRs [14]. A safety signal is defined by an ADR that exceeds what would be expected to be associated with a product's use based on what has been previously reported in past clinical trials [14]. Detection of these safety signals in a timely manner is an important aspect of PV. PV is a multidisciplinary science and is specifically important to “clinical medicine, clinical and pre-clinical pharmacology, immunology, toxicology and epidemiology” [15]. This broad scope underscores PV's critical role in drug safety.

The most traditionally used dataset in PV for ADR detection utilizes a Spontaneous Reporting System (SRS) database, such as the FDA Adverse Event Reporting System (FAERS) and WHO’s VigiBase [16]. Spontaneous reporting is the process of recording and reporting clinical observations of a suspected ADR with a marketed drug [15]. While this is the most traditional method of data collection in PV, it does pose some flaws, such as reporting bias, poor documentation, and lower reporting rates for older products [16]. These flaws can lead to underreported ADRs, which is not ideal for patient safety.

Other PV data sources for ADR detection include published case reports, cohort studies, and post-marketing clinical trials [15]. Like spontaneous reporting systems, these methods come with their own limitations. For example, using published case reports comes with the risk of gathering data from a few published cases, since only a very small proportion of cases are published, as well as the possibility of delays between the occurrence of events and the publication itself [15]. Despite the diversity of data sources used in PV, limitations in these methods can result in missed or underreported ADRs.

After ADR data have been collected, they need to be analyzed to detect certain patterns and risks of pharmaceutical drugs and their adverse effects. One primary method that achieves this goal is through a process that mines through data from various reporting systems, such as FAERS. This process generates safety signals that create hypotheses for further investigations [17]. This method has proven to be quite successful, as FAERS has successfully identified previously underreported ADRs and contributed to more than 50% of all post-market safety-related label changes [17]. In addition to the more traditional analytical methods, other, newer methods are being used, such as the Sentinel System. The Sentinel System is utilized to enhance and extend the potential of PV by studying specific drug-event outcomes, which generate safety signals [17]. It does so by leveraging EHR systems and insurance claims data and has greatly improved PV efforts [17]. The Sentinel System differs from traditional methods because its analyses are submitted to partner networks and run independently at each site. The data are then combined to provide a more complete safety profile [17]. While these methods are essential for PV, they still have their limitations. FAERS case reports, for example, are limited by incompleteness, bias, and inconsistency, as well as not measuring the total number of exposures in the population, all of which lead to incomplete adverse event data [17].

Despite these limitations, PV remains a critical responsibility shared by healthcare providers, pharmaceutical companies, and patients to ensure the safe use of pharmaceutical drugs [14].

Artificial intelligence (AI)

AI is a branch of computer science focused on developing systems that can perform tasks typically requiring human intelligence, such as data analysis, pattern recognition, and decision-making. It has become an integral part of people’s personal lives and is now being incorporated into scientific research, the healthcare system, and PV [8]. PV, as discussed previously, is a complex science that requires the collection and analysis of large amounts of data from a variety of sources to detect ADRs. Processing and analyzing such vast data are highly complex and resource-intensive. It is almost like separating "needles from haystack" [8]. Given the increasing volume of data, there is growing interest in integrating AI technologies into PV systems [8].

Through different key methods, AI can have a significant impact on detecting ADR signals [1]. These specific features of AI include ML and natural language processing (NLP) [18]. ML techniques analyze structured data, such as adverse drug reports, by focusing on the learning aspect of AI to develop algorithms for pattern recognition [19]. NLP processes free text from sources such as EHRs to identify ADRs [9]. Using these new technological methods, such as NLP, can yield positive results for the effectiveness and accuracy of identifying ADRs [20]. Furthermore, pilot studies have shown that ML models show promise in detecting ADRs earlier than traditional methods [21]. Prior studies, research, and the use of ML and NLP through AI demonstrate the ability to enhance data collection and analysis of ADRs.

To better help understand and visualize how AI, through ML and NLP, can enhance PV, we consider some of the following examples.

We consider a scenario in which data are collected from a diverse patient population. These data include the patient’s demographics, current prescriptions, different diagnoses, etc. An AI ML algorithm is then trained on these datasets to identify certain patterns and possible risk factors associated with ADRs. Then, over a given period of time, this model will be able to predict which patients are more likely to experience an ADR from a certain medication, which can help inform and assist healthcare professionals in making the best possible decision for their patients and possibly prevent these patients from experiencing an ADR.

Another example that uses an NLP model can include extracting relevant patient data from EHRs. Healthcare professionals typically document patient information in free-text form in the patient’s EHR. An AI NLP system can scan through this free text to extract side effects and particular drugs administered or taken by the patient. For example, if the patient was given drug “X” and started experiencing “Y” symptoms, the NLP model can flag this interaction and designate it as a potential ADR. This NLP model will greatly enhance the rate at which ADRs are detected by automating the extraction of relevant clinical information from unstructured text.

Potential role of AI in pharmacovigilance

Through ML and NLP, AI has the potential to improve certain healthcare sciences, particularly PV. The volume of PV data has grown to a scale that makes manual analysis increasingly impractical [18]. AI can benefit PV by reducing cycle times through spontaneously processing information, improving the quality and accuracy of information, reducing the time of case processing, and managing diverse datasets [18].

To date, there have been multiple studies investigating the application of AI in PV, and they have shown some very promising results. One such study conducted by Dsouza et al. [22] systematically reviewed findings from 13 studies that incorporated ML to predict ADRs. This systematic review found that ML algorithms can benefit the prediction of ADRs by identifying drug-event associations more efficiently, reducing the manual processing of data, and that AI systems, such as NLP, can significantly reduce processing times, improve accuracy, and decrease the workload of PV systems and professionals [22]. Integrating ML and NLP into PV systems has been shown to improve the efficiency and accuracy of ADR detection by reducing reliance on manual data processing.

Another similar systematic review conducted by Sessa et al. [23] compared the performance of AI to traditional pharmacoepidemiologic techniques. They compiled 72 original articles, five reviews, and compared the performances of AI to traditional PV methods. At the end of their study, they concluded that in half of their comparisons, AI performed better than traditional PV methods, with high variety in the performances between different AI methods [23]. They do, however, also state that many of these techniques have hardly been researched and that more research is required on this subject matter.

Similarly, Salas et al. [24] evaluated 66 articles to evaluate the usefulness of AI in PV. After identifying these articles and conducting a systematic review, they found that, through ML, AI can efficiently identify safety signals [24]. They also found that AI is able to process and analyze large amounts of data and that ML models can enhance PV processes and produce more efficient ways to analyze data pertaining to safety [24].

Given the growing body of research and increasing adoption of AI models, the future of PV appears to be moving toward greater automation and enhanced data analysis capabilities [25].

Limitations

Despite the potential of AI to enhance PV, several limitations must be addressed. A key limitation is AI's dependence on the quality and completeness of available data. Inadequate or poorly curated datasets can lead to inaccurate ADR detection [26]. Training AI on datasets with missing or underreported ADRs can lead to inaccurate predictions and missed signals.

Ethical concerns are another limitation for incorporating AI into PV. Kumar et al. [27] wrote that the issue is the access of patient data without regulations and patient consent. This can lead to ethical and moral issues and violate the patient-physician relationship [27]. Ensuring the ethical use of sensitive patient data requires robust regulatory frameworks and clear consent protocols. If AI is to be incorporated into PV systems, there needs to be certain guidelines in place to ensure patient privacy is not broken and that there are no ethical violations.

Interpreting PV data is another limitation that needs to be addressed. ADR reports require multiple decision-making processes to occur. Desai [8] wrote that the evaluation of individual case safety reports (ICSRs) is not uniform or can be easily automated, as the clinical presentation and ADRs of patients often require human judgment [8]. Although AI surpasses traditional data processing methods in sophistication, human oversight remains essential for interpreting complex clinical data.

The lack of research in this area is another major limitation. Despite growing interest, there remains a lack of comprehensive research evaluating the long-term impact of AI integration into PV [28]. Dsouza et al. [22] stated that more research needs to be conducted to understand the long-term effects of integrating AI into PV [22]. Furthermore, a study previously discussed that was conducted by Sessa et al. [23] also emphasized the need for further research to be conducted, and it is necessary to compare AI to more traditional techniques [23]. While the prospect of incorporating AI into the world of PV shows promise, more conclusive studies on the effects of AI in PV need to be conducted before widespread adoption can be justified.

## Conclusions

Modernizing PV through AI integration could significantly benefit both healthcare providers and patients by improving data collection and analysis. Through ML and NLP, AI has the potential to collect, process, and analyze data at an unrivaled rate, enhancing data analysis speed, predictive accuracy, and the overall efficiency of ADR detection. Although limitations exist, combining AI with human expertise may help mitigate these challenges. With the exponential rise in technology we have seen over the last decade, it is crucial that data-intensive sciences such as PV incorporate AI to enhance their capabilities.
